# A simple, cost-effective emitter for controlled release of fish pheromones: Development, testing, and application to management of the invasive sea lamprey

**DOI:** 10.1371/journal.pone.0197569

**Published:** 2018-06-13

**Authors:** C. Michael Wagner, James E. Hanson, Trevor D. Meckley, Nicholas S. Johnson, Jason D. Bals

**Affiliations:** 1 Department of Fisheries and Wildlife, Michigan State University, East Lansing, Michigan, United States of America; 2 Department of Chemistry and Biochemistry, Seton Hall University, South Orange, New Jersey, United States of America; 3 USGS, Great Lakes Science Center, Hammond Bay Biological Station, Millersburg, Michigan, United States of America; Laboratoire de Biologie du Développement de Villefranche-sur-Mer, FRANCE

## Abstract

Semiochemicals that elicit species-specific attraction or repulsion have proven useful in the management of terrestrial pests and hold considerable promise for control of nuisance aquatic species, particularly invasive fishes. Because aquatic ecosystems are typically large and open, use of a semiochemical to control a spatially dispersed invader will require the development of a cost-effective emitter that is easy to produce, environmentally benign, inexpensive, and controls the release of the semiochemical without altering its structure. We examined the release properties of five polymers, and chose polyethylene glycol (PEG) as the best alternative. In a series of laboratory and field experiments, we examined the response of the invasive sea lamprey to PEG, and to a partial sex pheromone emitted from PEG that has proven effective as a trap bait to capture migrating sea lamprey prior to spawning. Our findings confirm that the sea lamprey does not behaviorally respond to PEG, and that the attractant response to the pheromone component was conserved when emitted from PEG. Further, we deployed the pheromone-PEG emitters as trap bait during typical control operations in three Great Lakes tributaries, observing similar improvements in trap performance when compared to a previous study using mechanically pumped liquid pheromone. Finally, the polymer emitters tended to dissolve unevenly in high flow conditions. We demonstrate that housing the emitter stabilizes the dissolution rate at high water velocity. We conclude the performance characteristics of PEG emitters to achieve controlled-release of a semiochemical are sufficient to recommend its use in conservation and management activities related to native and invasive aquatic organisms.

## Introduction

Biological invasions precipitate costly changes to ecosystem structure and function that accrue over decades and not all invaders are equally harmful. When society deems the damage caused by an established invader of sufficient concern to attempt control, long-term maintenance management (vs. eradication) is frequently the only viable option [1–2]. This is particularly true in aquatic ecosystems where control of invasive species is made problematic by the size, complexity, and openness of the habitats, and the typically large number of invaders (e.g. >180 invasive species in the North American Great Lakes) [3]. Because the management of aquatic invasive species occurs across large spatial and ecological contexts, often in ecosystems experiencing multiple environmental stressors, there is considerable desire for benign control tactics that do not interfere with achieving broader ecosystem management goals [4]. One such approach, the application of species-specific semiochemicals that induce attraction (e.g. sex pheromones) or repulsion (e.g. alarm cues) to guide undesirable species into areas targeted for control, has proven useful in the management of terrestrial insect pests [5]. Fishes, one of the most important groups of invaders in freshwater and marine ecosystems, also rely extensively on olfactory cues to mediate behaviors critical to foraging, reproduction, and the evasion of predators [6–9]. Thus, the manipulation of semiochemical information should prove useful to achieving the management of invasive fishes in many aquatic ecosystems [10–12].

The process of developing a semiochemical control tactic requires the isolation, identification, and synthesis of each active constituent in the odor, and the elucidation of mixtures that elicit persistent behavioral responses [13]. Consequently, any deployment technique must reliably deliver the proper compounds in the proper ratio over sustained periods to ensure the efficacy of the application is maintained. In addition, the emitter matrix should not chemically alter the semiochemical, should protect the substance from sources of environmental degradation prior to release (e.g. ultraviolet light and oxygen), and should be environmentally benign and/or rapidly degrade in the environment [14–16]. Although there has been substantial testing of semiochemical emitters for highly volatile air-borne compounds [16], little attention has been paid to the development of emitters that meet these criteria and are designed to function in natural waters.

Polymers, widely used for the controlled release of various active agents—pharmaceuticals, fertilizers, perfumes, and many more [17–19]–represent a class of material likely to be useful as a controlled release matrix in water. For example, polyethylene glycol (PEG) [20] and other neutral polymers such as polyvinyl alcohol (PVA) [21] and starch [22] act by slowly dissolving in the desired medium, releasing the active agent mixed into the polymer. Depending on the compatibility of the materials, the agent is either dissolved in the polymer (dispersed molecularly) or thoroughly mixed with the polymer (dispersed as small crystals or droplets). The rate of polymer dissolution, and therefore release, depends on the type and state of solvent (e.g. temperature, pH and ionic strength), and the molecular weight of the polymer. For applications in natural aquatic ecosystems, it is critically important to choose polymers that are nontoxic and biodegradable. The PEG polymers are widely used in many commercial products (shampoos, cosmetics, etc.) and are known to degrade relatively quickly in the environment [22–24]. PVA is also known to biodegrade, and starch is a natural polymer that is rapidly broken down in nature [22]. The release rate from these polymers is straightforward to regulate, as the active agent release essentially follows polymer dissolution, and the rate of polymer dissolution can be modulated by changing the molecular weight of the material and/or design of the device. Here, we report the results of the development and testing of a simple polymer emitter for the release of fish pheromones in water to achieve invasive species control.

Our test system involved the sea lamprey (*Petromyzon marinus*), an invasive fish that parasitizes other fishes of considerable societal value in the North American Great Lakes and is subject to an international control program costing in excess of $20 million (US) each year [25]. The landlocked sea lamprey is a potamodromous organism, returning to rivers to spawn and die after feeding in the lakes as a parasite for 12–18 months. During the return migration, the sea lamprey does not home to natal streams [26–27]. Rather, it responds to the emission of odors from stream resident larval sea lamprey, and other lamprey species, to find suitable spawning habitat [28–30]. Upon arrival at the spawning grounds and sexual maturation, the male begins to release a multi-component sex pheromone that attracts ovulating females to his nest to complete reproduction [31]. Several constituents of the larval odor [32–33] and the male sex pheromone [34] have been isolated and chemically characterized. Among these, only the sex pheromone component 3-keto petromyzonol sulfate (3kPZS) has been successfully synthesized [35], tested in control applications [36], and registered for use in pest management in the Great Lakes [37]. Specifically, 3kPZS is effective in luring ovulating females into traps baited with the odorant [35], and can increase upstream movement and attract sexually immature migrants in certain circumstances [38–40], though the mechanism operating prior to sexual maturation is unknown. Our goal was to develop a cost-effective and environmentally benign polymer-based emitter for deployment of 3kPZS as a sea lamprey control tool. We first evaluated the dissolution behavior of several polymer candidates in the laboratory (e.g., dissolution rate, release rate, realized 3kPZS concentration, etc.), and selected PEG with a molecular weight of 6000 based on its performance (see S1 Text and S1 Fig for a description of the selection process). We then tested the emitter by: (Objective 1) ascertaining whether migratory sea lamprey respond to the polymer in the laboratory; (Objective 2) ascertaining whether the 3kPZS-impregnated polymer elicits a sufficiently strong behavioral response from ovulatory female sea lamprey in a natural stream; (Objective 3) testing the application of the 3kPZS emitter during routine sea lamprey trapping operations in a natural stream; and (Objective 4) designing and testing a deployment apparatus to control the polymer dissolution rate in the turbulent flowing waters typical of rivers.

## Materials and methods

### Objective 1: Behavioral response to PEG6000 in the laboratory

To ascertain whether sea lampreys were repelled by, attracted to, or indifferent to the presence of PEG6000 dissolved into water, we evaluated space-use in a laboratory flume via two-choice testing per the methods of Wagner et al. [41]. During the experiment, two emitter tubes were placed at mid-water, oriented perpendicular to the flow, at the upstream end on one side of a raceway. One contained the PEG6000 polymer, activating approximately half of the discharge with PEG6000, and the other was empty (control). Free-swimming migratory-phase female sea lampreys were monitored as the polymer dissolved to determine whether they were attracted to the PEG6000 (spent significantly more time on the PEG6000 side vs. the control side), were repulsed by the PEG6000 (spent less time on the emitter side), or exhibited no preference. Their space use was compared to a pre-trial period of activity when the emitters were not present.

Sub-adult female sea lampreys were captured during the spring spawning migration in two tributaries to Lakes Huron (Cheboygan and Ocqueoc Rivers) by the U.S. Fish and Wildlife Service (USFWS). Subjects were initially sorted by sex at the point of capture and were transported daily to the HBBS holding facilities. Upon arrival at the station, sea lampreys were transferred into several 400-gallon holding tanks at a maximum density of 50 individuals per tank. Each tank was continuously aerated with regenerative blowers and received continuous flow of water from Lake Huron, fully exchanging the water in the tank every 1.5–2 h. The water temperature in the holding tanks typically increases from 5° C when trapping operations begin in early May to 18° C when operations cease in late July [42]. All handling and care procedures were approved by the Michigan State University Institutional Animal Care & Use Committee prior to the start of the experiment per permit number AUF 02-11-027-00.

**Fig 1:**
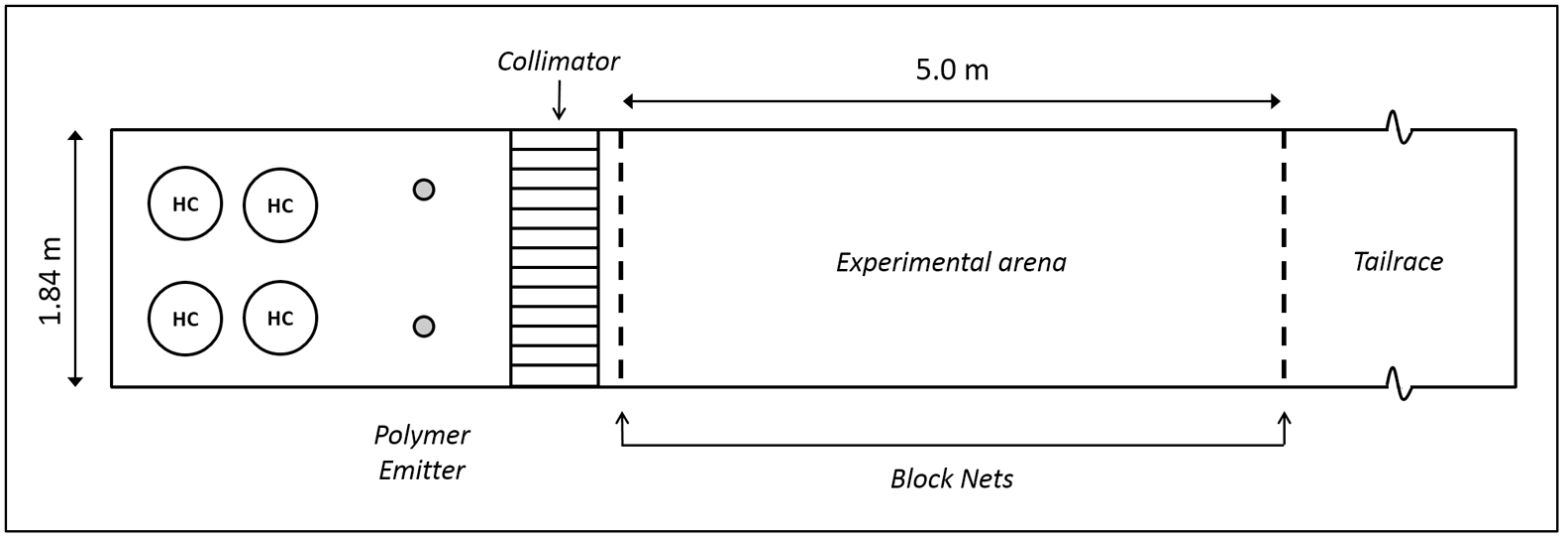
Schematic of the laboratory raceway setup for the behavioral assay. Water flow is left to right in the image. Four groups of ten female lampreys were held upstream of the polymer emitter in holding cages (HC). At the start of the trial a group is moved into the experimental arena and released. During each trial a single emitter was used, alternating from right to left sides of the channel across trials. A video camera above the experimental arena recorded movements.

Eight replicate trials took place on a single night in two linear 20 m concrete laboratory raceways (four trials per raceway) at the HBBS (Fig 1). The raceways received a slow, continuous flow of water pumped directly from Lake Huron, and experienced a natural day-night light schedule over the course of the experiment (24 May 2011). The discharge in each raceway was set to 0.001 m^3^ sec^-1^, with corresponding velocities of 1.35–1.43 cm sec^-1^ depending on location. The experimental section of each raceway was 5.00 m long and 1.84 m wide with a water depth of 40 cm. A single infrared-sensitive video camera equipped with two infrared light arrays was mounted directly over the experimental section of each raceway. Sea lamprey movements were observed in an adjacent room on video monitors and recorded onto digital media for later analysis.

Behavioral trials began approximately one hour after the time of sunset (22:00 h). Six hours prior to the start of the trials, eight groups of ten female sea lampreys each were moved into separate holding cages placed upstream of the experimental section (four cages per raceway). As female sea lampreys do not exhibit behavioral responses to the odor of other females, holding subjects upstream does not affect the behavior of lampreys in the experimental arena [43]. At the start of a trial, a single cage was carefully and slowly moved into the middle of the experimental arena and lifted to release the sea lampreys. Each experimental trial was 40 min long and consisted of a 20 min pre-stimulus period and a 20 min stimulus period during which the PEG6000 emitter was introduced into one side of the raceway. We constructed the PEG6000 emitters from female-female PVC couplers (1.91 cm inside diameter (ID), 5.1 cm L) filled with 14.6 ml PEG6000 (Alfa-Aesar, Ward Hill, MA). This volume was expected to last eight hours in a natural stream. We alternated the side of the raceway receiving the stimulus across replicates within each raceway such that four trials were completed with the emitter on the right side, and four trials with the emitter on the left side. To test the hypothesis that PEG6000 did not induce attraction or repulsion, we compared the proportion of sea lampreys on the stimulus side of the raceway during the pre-stimulus and stimulus periods via a two-tailed paired t-test. The proportions were ascertained by counting the number of lampreys on the stimulus side at 30 sec intervals only during the final 10 min of the pre-stimulus and stimulus periods (to avoid any temporal autocorrelation effects of adjacent time periods of observation). The proportions were arc-sin square root transformed prior to analysis and met the assumptions for hypothesis testing (Shapiro-Wilk test, W = 0.95, P > 0.05).

### Objective 2: Behavioral response to 3kPZS emitted via PEG6000 in a natural stream

To ascertain whether 3kPZS emitted by PEG6000 (hereafter termed 3kPZS emitter) is attractive to free-swimming sexually mature female sea lampreys, we performed a pair of preference tests. First, we tested whether 3kPZS emitted from the polymer is attractive to ovulating females by comparing it to a control (a blank polymer emitter). Second, we tested whether 3kPZS emitted from the polymer is as attractive as a pumped mixture of 3kPZS eluted in methanol and water used in previous experiments (e.g. [35, 44]). We hypothesized 3kPZS emitted from a polymer will be highly attractive to ovulating females and predicted: (1) significantly more sea lampreys will approach the 3kPZS-impregnated polymer emitter vs. the polymer alone or neither emitter (preference test #1), and (2) there will be no significant difference in the number of sea lampreys that choose to approach the 3kPZS emitter vs. the pumped mixture of 3kPZS and methanol (preference test #2).

We constructed the 3kPZS emitters as per Objective 1. A stock solution of 3kPZS (>98% pure, Bridge Organics, Inc.) was prepared in methanol with a concentration of 5 mg ml^-1^. PEG6000 was melted in a 70 °C water bath and a volume of the 3kPZS stock solution was added to the melted polymer to create emitters that would release 3kPZS at a minimum rate of 1 mg h^-1^, a concentration that has proven behaviorally reactive in prior sea lamprey studies [35, 43]. To ensure this minimum, for each 7.3 ml of PEG6000, 1 ml of the 3kPZS stock solution was added: thereby, a single 14.6 ml 8 h emitter was provisioned with 10 mg of 3kPZS. The PEG6000-3kPZS mixture was allowed to stand at 70 °C for 6 hours to allow the methanol to fully evaporate. The coupler tubes were prepared for filling by placing them on a metal plate (usually cooled by laying it across a bed of ice). Each tube was sealed to the plate by a small amount of pure PEG6000, melted and poured onto the plate for that purpose. The PEG6000-3kPZS mixture was then poured into the couplers to fill them to the top. Some volume shrinkage was observed upon cooling. Once the filled tubes had hardened completely, they were removed from the plate and ready for use. 3kPZS batch # 183-EJH-290-3 synthesized during February 2010 (Bridge Organics, Vicksburg, Michigan) was used. This 3kPZS batch had a purity >99% based on high pressure liquid chromatography and mass spectrometry (reviewed by NSJ). Application of the emitters in field-testing was approved by the U.S. Environmental Protection agency via the Experimental Use Permit provisions of the Federal Insecticide, Fungicide, and Rodenticide Act (permit #5437-EUP-4).

**Fig 2:**
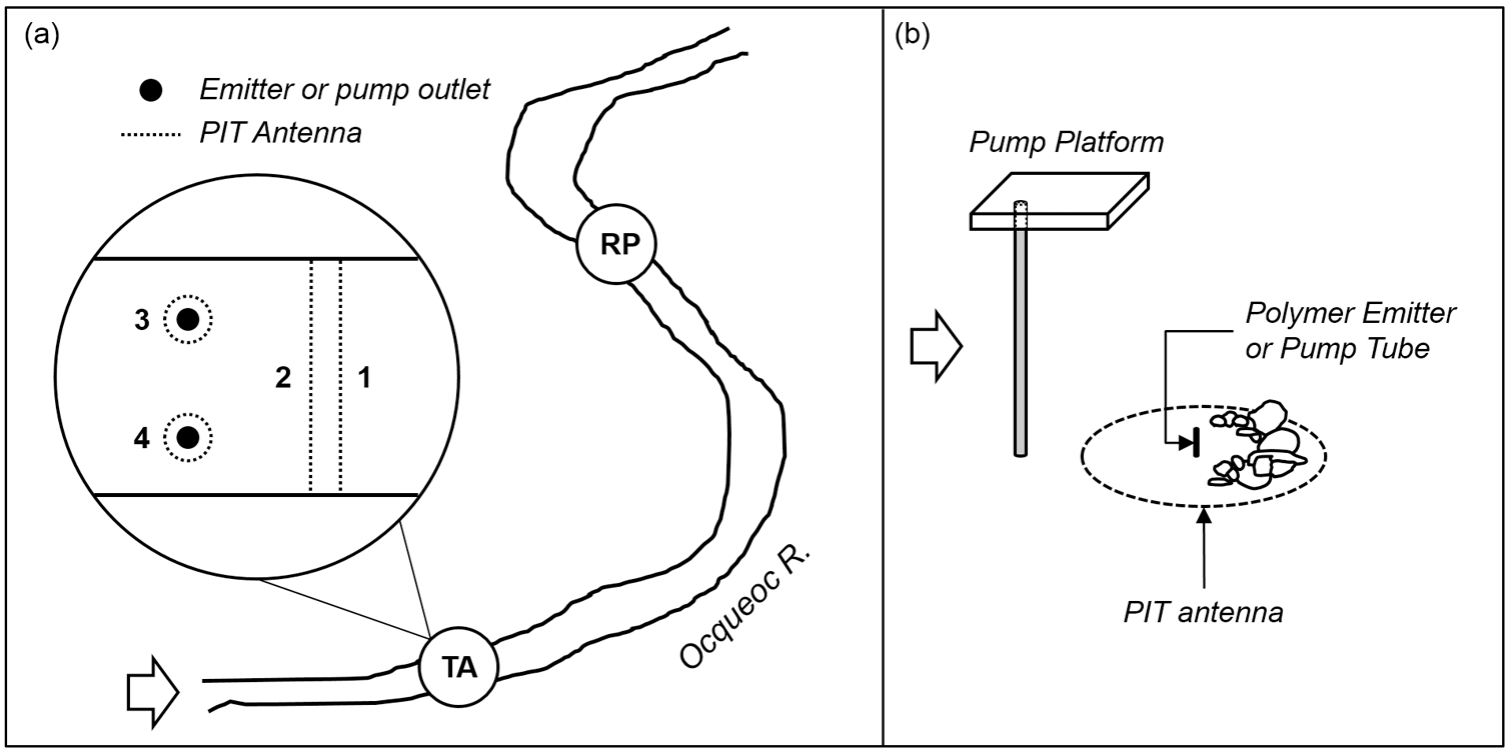
A schematic of the Ocqueoc River field site. (a) Lampreys were held in cages at the release point (RP) until the start of the trial. The upstream test area (TA) was equipped with four PIT antennas; two to detect upstream movement into the area (#1 and #2), and one each surrounding the point of emission of 3kPZS, either by polymer emitter or pump tube, to detect attraction (#3 and #4). (b) Detail of the 3kPZS emission apparatus. The PIT antenna surrounded a simulated lamprey nest. At the center of the nest either a PEG6000 emitter was placed (both tests), or the end of a buried tube emerged from the sediment (Test #2 only). When used, the 3kPZS-methanol mixture was pumped from a carboy placed on the platform. In both diagrams the white arrows indicate the direction of water flow.

The preference tests were performed at a long-term experimental site in the Ocqueoc River, MI (Presque Isle County, Michigan, USA) used previously to examine sea lamprey migratory behavior in rivers (e.g. [30]). In the test area (Fig 2a), we constructed two sea lamprey nests from pebbles and cobbles in the stream at points representing approximately one- and two-thirds the width of the stream (Fig 2b). Each nest was surrounded by a flat Passive Integrated Transponder (PIT) antenna of 0.75 m diameter, and two additional stream-width PIT antennas were placed 15 m downstream to detect initial approach of tagged sea lamprey to the test area. We constructed wooden platforms above each nest to hold the carboy, pump, battery, and PIT antenna modules. Either the 3kPZS emitter, or the tube emitting pumped pheromone, was placed in the center of a nest. The 3kPZS emitter was attached to a small clear plastic stake with a rubber band at a height of 5 cm above the substrate. The pump tube was buried from the base of the platform stand to the center of the nest, where it emerged to a height of 5 cm above the substrate.

Each subject used in the field experiment was fitted with an internal 23-mm PIT tag (Oregon RFID). We selected female sea lampreys in good condition and moved them temporarily into holding cages placed in the Ocqueoc River downstream of our study site to facilitate sexual maturation. Stream water temperatures ranged from 15–22°C during the holding period. We visually assessed each lamprey for signs of maturation (release of gametes upon gentle squeezing of the abdomen) according to the procedures described by Siefkes et al. [43]. Sexually mature females were immediately returned to the station and held for 1–2 days until needed. Prior to use in the experiment, each subject was measured (TL, cm) and weighed (g). Because mature female sea lampreys have thin-walled abdomens after the onset of ovulation, subcutaneous and intraperitoneal tag implantations are problematic and inefficient (i.e. tag loss and injury are frequent). We sutured a small piece of polyethylene tubing containing a PIT tag onto the mid-dorsum of each subject, oriented longitudinally, by suturing either end to the epidermis (suture: size 3–0, Ethicon Inc., Cornelia, Georgia, USA). No anesthetic was applied during tagging as common fish anesthetics (e.g. MS-222) are known to disrupt olfactory-mediated behaviors [45–46]. This technique has proven highly effective in studies with ovulating sea lamprey [40, 44, 47]. After tag attachment, each subject was observed for 30 min to ensure no adverse event had occurred. All handling, care, and tagging procedures were approved by the Michigan State University Institutional Animal Care & Use Committee prior to the start of the experiment per permit number AUF 02-11-027-00.

Eight hours prior to the start of a trial, we placed a group of 13–29 PIT-tagged ovulating female sea lampreys (depending on availability) into a holding cage 100 m downstream of the emitters to ensure acclimation to stream conditions. Two hours before the start of a trial an emitter or pump tube was placed on either side of the stream to ensure adequate dispersion of the odorant and/or polymer. The subjects were released near sunset and their movements recorded for four hours. At the conclusion of each trial, we downloaded the PIT-tag detections onto a waterproof Meazura PDA. Recorded information included the identification number, dates and times of signal reception, and the direction of movement for each detection event. Several abiotic variables were measured prior to each trial including mean velocity and discharge, water temperature, and general weather conditions. The distribution of approaches to the PEG6000 blank emitter, 3kPZS-PEG6000 emitter, or neither (Preference Test #1) was examined with a chi-square test of equal proportions (N = 51). Similarly, the distribution of approaches to the 3kPZS-PEG6000 emitter, pumped 3kPZS, or neither was examined with a chi-square test (N = 48). In both cases approaches were determined by the first hit on the PIT antenna surrounding a nest, and ‘neither’ was assigned if the lamprey entered the Test Area (i.e. was recorded on PIT antennas 1 then 2, in sequence, per Fig 2a) but failed to hit on either nest antenna. The null expectation was equal proportions within each group, as each nest PIT antenna covered approximately one third of the channel width at the test site.

### Objective 3: Efficacy of PEG6000 3kPZS emitters during trapping operations

To ascertain whether 3kPZS released via PEG6000 increased catch of at-large sea lamprey in traps operated by the USFWS during the return migration [48], as has previously been reported for pumped 3kPZS [35], 3kPZS emitters were used to bait traps located at dams in three Michigan streams during 2012 (Cheboygan, Manistique, and Muskegon Rivers). The Cheboygan, Manistique, and Muskegon Rivers are relatively large rivers for the Great Lakes basin with average discharges ranging 20–100 m^3^ sec^-1^ and widths up to 100 m. The traps are traditionally operated as passive devices (i.e. without bait) that are oriented to the local flow and rely on repeated encounters with the entrance funnel as the lamprey attempts to pass the dam [49]. The Cheboygan and Manistique Rivers have two fish traps placed about 1 m apart. Therefore, to test if 3kPZS emitters increased catch in the Cheboygan and Manistique Rivers, sea lamprey control agents placed a 3kPZS emitter into one of the paired traps during the natural migration, alternating which trap of the pair received the emitter on subsequent nights. The USFWS operates a single trap on the Muskegon River; consequently, the single trap was baited with 3kPZS on alternating nights. The experiment was conducted from May 3^rd^ to June 9^th^ in the Cheboygan River, May 5^th^ to June 8^th^ in the Manistique River, and May 8^th^ to June 3^rd^ in the Muskegon River.

3kPZS emitter design was based on data obtained from the dissolution and behavioral experiments describe above. The goal was to release 3kPZS at a rate of 2 mg h^-1^ for 10 hours each night. This rate was approximately double the rate used for the field behavior experiment (Objective 3), and previously reported field studies with 3kPZS in small discharge (~ 1 m^3^ sec^-1^) streams [35, 38, 43]. To achieve this target, 20 mg of 3kPZS was added to 18.3 g (± 0.3, SD) of molten PEG6000. The molten mixture was poured into a cross-linked high-density polyethylene tube (PEX) measuring 8.5 cm long with an inside diameter of 1.2 cm (outside diameter 1.5 cm). 3kPZS emitters were placed in an automatic pet feeder (QPets 4 Meal LCD Automatic Pet Feeder) programed to drop a single emitter at 2100 h into a mesh bag located in the trap where the water flow through the trap was ~ 20 cm sec^-1^. The mesh bag was used to suspend the emitter upstream of the trap entrance and to reduce turbulence around the emitter to maintain a steady dissolution rate throughout the study. 3kPZS batch # 183-EJH-290-3 was also used in this study, with use of the emitters approved by the US EPA via Experimental Use Permit #5437-EUP-4.

To determine if sea lampreys were more likely to be captured in traps when baited with 3kPZS, and if the response varied among streams, a generalized linear model (binomial, link identity, R Core Team 2014 per [38]) was used to model if variability in nightly trap catch was explained by if the trap was baited and what stream it was in. Further, trap efficiencies, defined as the proportion of the run captured by the traps during the entire year, have been estimated historically via mark-recapture models [48] for each river. We compared the trap efficiency observed in 2012 to all other years when 3kPZS was not applied using box and whisker plots, predicting an increase in trap efficiency in 2012 akin to that reported by Johnson et al. [38] in other Michigan streams.

### Objective 4: Test of an emitter housing to control dissolution rate in turbulent flow

**Fig 3:**
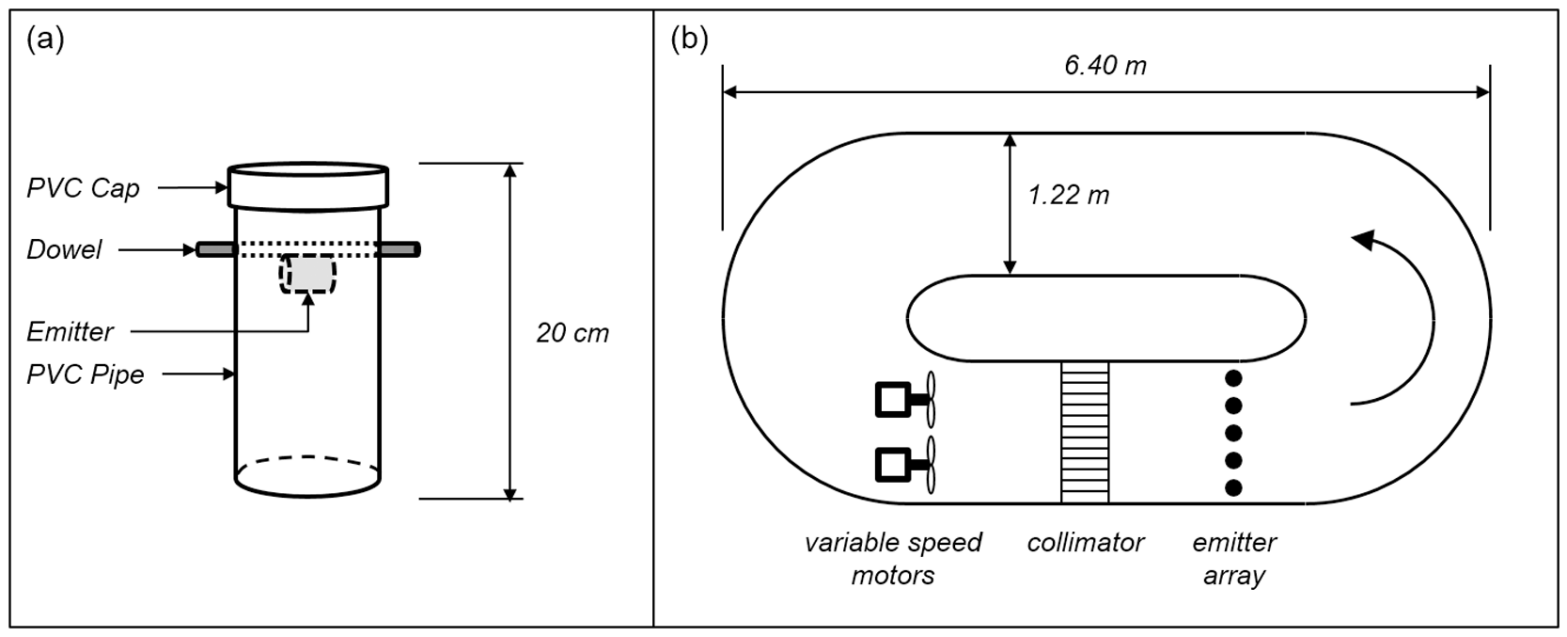
Emitter housing and test tank used during Objective 4. (a) Design of the emitter housing. The emitter was attached to the dowel with a single rubber band. The housing was mounted in the flow tank as shown, with the bottom open to the water. (b) Schematic of the recirculating flume tank. Flow was maintained with two trolling motors mounted upstream of a PVC collimator. During each trial five emitters were mounted at mid-depth and equidistant across the channel.

During the field experiments (Objectives 3 and 4), examination of partially dissolved emitters indicated the effects of natural turbulence on dissolution rate. Specifically, pits and peaks appeared in the surface of the polymer, increased the exposed surface area, and led to more rapid dissolution in high velocity circumstances. In a recirculating flume tank, we tested whether placing the emitter in a housing (a segment of large diameter pipe capped on one end) would reduce both the rate of dissolution and the variation in dissolution rate across multiple similar emitters vs. emitters mounted in the flow without the housing. The housing (Fig 3a) was constructed from a 0.2 m length of 7.62 cm ID PVC pipe with a cap placed on one end (without glue). A wooden dowel (0.61 cm dia.) ran through holes drilled in opposite sides of the pipe 5 cm below the cap, and the emitter was attached to the dowel with a single rubber band. We tested ten replicates of each emitter configuration (housed or exposed) at six velocities (0, 5, 10, 25, 50 and 100 cm sec^-1^) for 2 h. During a trial, five emitters were mounted equidistant across a straight section of the flume (Fig 3b), and two trials were completed for housed and exposed emitters. Emitter construction was identical to those used in Experiment #2, with no 3kPZS added to the PEG6000. Flow was maintained in the flume with two variable speed trolling motors (Minn Kota, Inc.) mounted at the upper end of the channel. Water depth was maintained at 66 cm, with the motors placed at mid-depth and the emitters mounted 25 cm above the substrate. Between the motors and the emitters, we placed a collimator constructed from 60 cm lengths of 1.91 cm dia. PVC pipe, glued into a block that filled with width and depth of the channel. The collimator partially smoothed the flow downstream of the motors to create flow more typical of a stream (i.e. without large vertical vortices). Each emitter was uniquely labeled and weighed before and after the trial to estimate the rate of polymer mass loss. We examined the fixed effects of emitter type (factor, housed or exposed) and observed mean water velocity (continuous) on the rate of observed mass loss (g polymer h^-1^) with a generalized linear model (Gaussian, link identity) via STATA 14.2 (STATA Corp.).

## Results

### Objective 1: Behavioral response to PEG6000 in the laboratory

**Fig 4:**
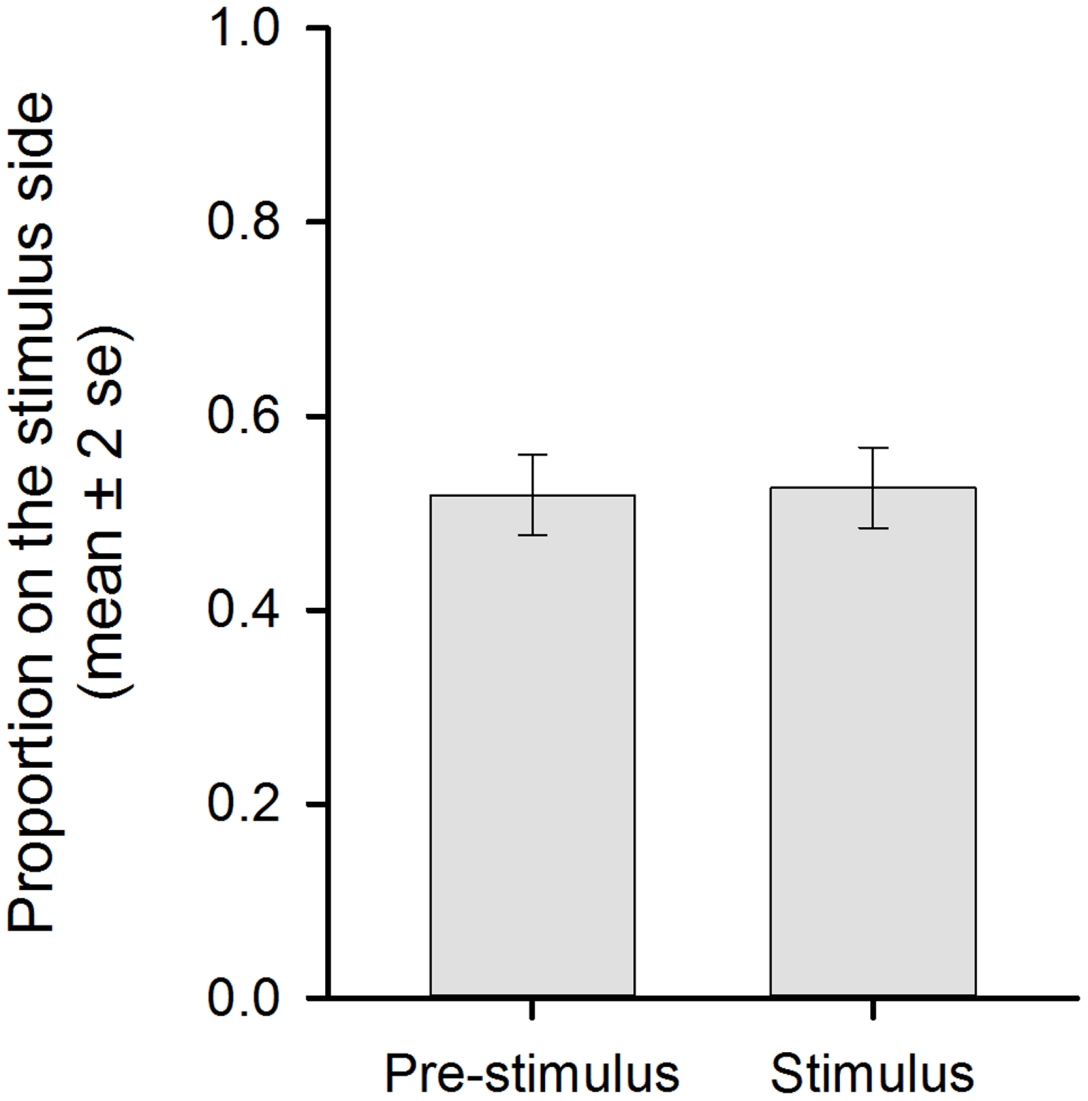
Migratory sea lamprey do not respond to the odor of PEG6000. Dissolution of PEG6000 (stimulus) into one side of the laboratory raceway did not alter the lateral distribution of sea lamprey vs. the pre-stimulus period (paired t-test, n = 8, P = 0.69).

During the laboratory trials, sea lampreys were neither attracted to nor repelled by PEG6000 (Fig 4). The proportion of lampreys on the stimulus side during PEG6000 emission did not differ from the pre-stimulus period (*t*_7_ = -0.41, P = 0.69).

### Objective 2: Behavioral response to 3kPZS emitted via PEG6000 in a natural stream

**Fig 5:**
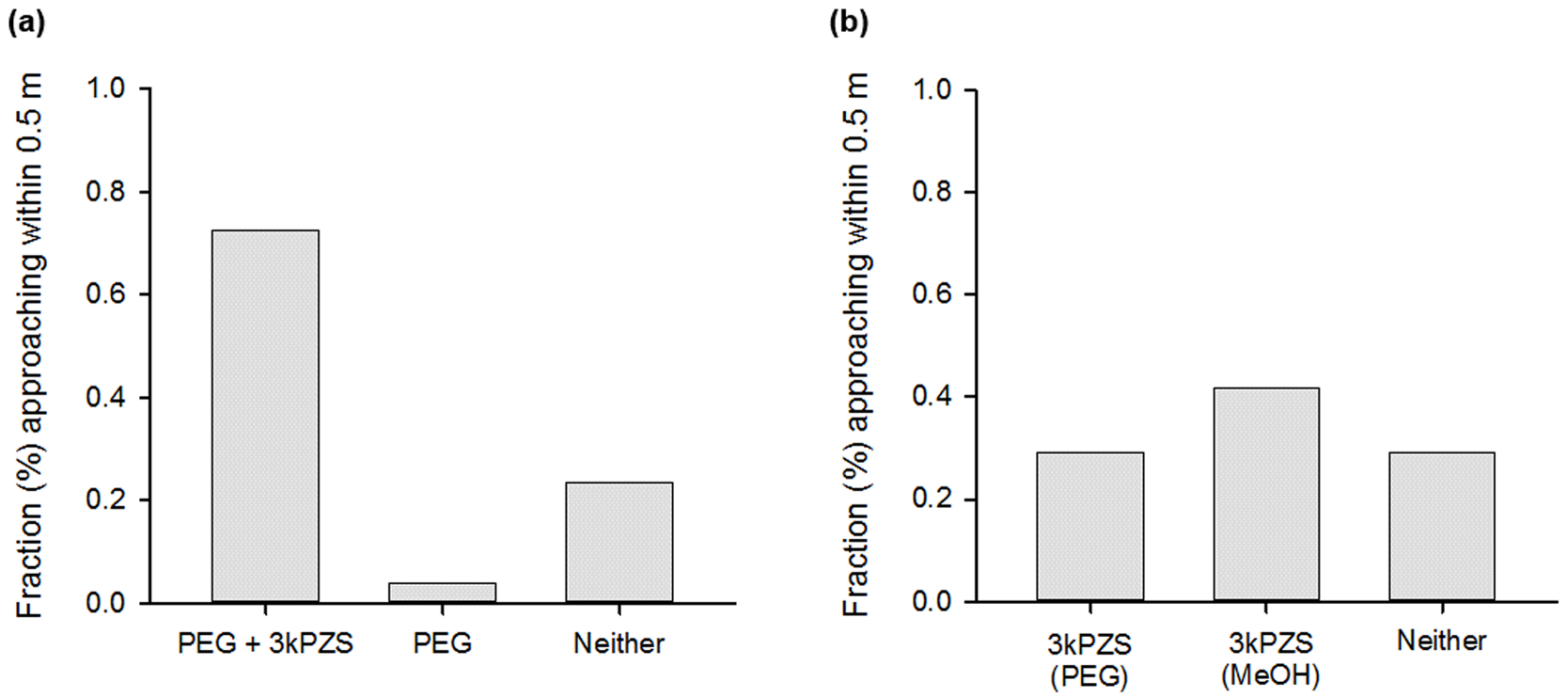
Results from the field behavioral experiments for Objective 2. (a) Preference Test #1. Ovulated female sea lampreys were significantly more likely to approach a PEG6000 emitter releasing the pheromone 3kPZS vs. a blank PEG6000 emitter or neither (χ^2^_2,51_ = 38.235, P<0.001). (b) Preference Test #2. Ovulated female sea lampreys did not prefer 3kPZS emitted from a PEG6000 emitter or pumped as a liquid in methanol and water (χ^2^_(2,48)_ = 1.50, P = 0.447).

When given a choice between PEG6000 and PEG6000 impregnated with 3kPZS (preference test #1), ovulating sea lampreys were significantly more attracted to the emitter releasing the pheromone (73%) vs. the blank emitter (4%) (χ^2^_2,51_ = 38.235, P<0.001, Fig 5a). Twenty three percent of the lampreys that entered the test area were not detected on either antenna. When given a choice between polymer-emitted and pumped 3kPZS (preference test #2), 67% of the lampreys approached an emitter after entering the test area. The lampreys exhibited no preference for pumped or PEG-emitted pheromone (χ^2^_(2,48)_ = 1.50, P = 0.447, Fig 5b).

### Objective 3: Efficacy of PEG6000 3kPZS emitters during trapping operations

In all cases but one (n = 91), the 3kPZS emitters were fully dissolved when the trap was checked the next morning between 0800 and 1000 (11 to 13 hours soak time). The feeder failed twice at the Cheboygan River, four times at the Manistique River, and twice at the Muskegon River, either because the emitter jammed in the feeder exit or the timer was not set correctly. Data from nights when the feeder failed were not included in the paired trap catch analysis. Discharge in the Cheboygan, Manistique, and Muskegon Rivers varied from 20–43, 13–22, and 50–170 m^3^ sec^-1^, respectively, during the trapping season. Given a release rate of 2 mg h^-1^ in each stream, the final fully mixed in-stream Molar concentration of 3kPZS applied to the Cheboygan, Manistique, and Cheboygan rivers varied from 2.4–5.6 x 10^−14^, 5.1–8.7 x 10^−14^ and 2.3–5.1 x 10^−14^ M, respectively. USFWS trap operators who applied 3kPZS with pumps during previous field tests [38] reported the emitters were simpler and faster than pumps, only taking about 1 minute to set-up per day.

**Fig 6:**
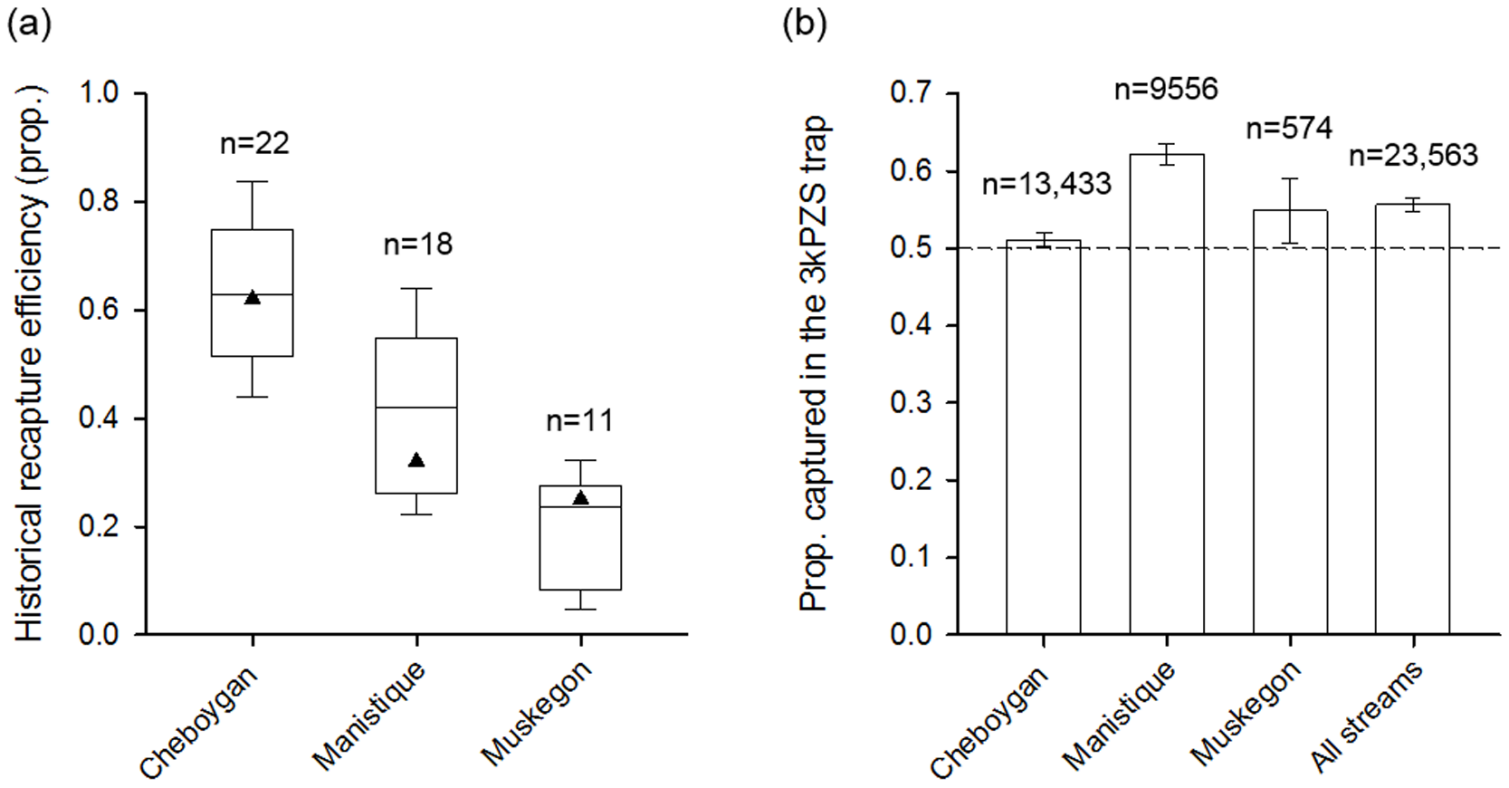
Performance of the emitter-baited traps (vs. a paired unbaited trap) during sea lamprey control operations. (a) Box and whisker plots of historical trap efficiency (proportion of the run captured) for each stream as estimated by mark-recapture (data obtained from P. Hrodey, USFWS, outliers omitted). The black triangle indicates the observed trap efficiency during 2012 when 3kPZS-PEG6000 emitters were applied, “n” indicates the number of years historical trap efficiency was estimated. (b) The proportion of the total catch that was captured in the 3kPZS emitter-baited trap with 95% binomial confidence intervals, “n” refers to the total number of sea lamprey captured in baited and unbaited traps during 2012. The dashed line indicates the null expectation of equal catch rate between the paired traps if the 3kPZS-PEG6000 emitter did not influence capture rate (which trap received the bait was alternated on subsequent nights).

Among all the streams, 13,678 pre-spermiated males were captured (7,514 in 3kPZS-baited traps or 55%), and 181 spermiated males were captured (101 in 3kPZS-baited traps or 56%). 9,632 pre-ovulatory females were captured (5,475 in 3kPZS-baited traps or 57%), and 28 ovulated females were captured (21 in 3kPZS-baited traps or 75%). The overall yearly trapping efficiency in the Cheboygan, Manistique, and Muskegon rivers during 2012 when 3kPZS PEG emitters were applied to one of the paired traps each night was near historical averages (Fig 6a). Traps baited with 3kPZS-PEG6000 emitters captured more sea lampreys than paired traps not baited with emitters (*X*^*2*^_(1, 23516)_ = 346.9, P < 0.001, Fig 6b). The 3kPZS-baited trap in each trap pair captured 50–63% of the total catch (Fig 6b), with increases of 4%, 65%, and 22% in the Cheboygan, Manistee, and Muskegon rivers, respectively (calculated as the baited trap catch minus the unbaited trap catch divided by unbaited trap catch per Johnson et al [38]). The improvement in capture success differed significantly among streams (*X*^*2*^_(2, 23514)_ = 92.2, P < 0.001).

### Objective 4: Test of an emitter housing to control dissolution rate in turbulent flow

**Fig 7:**
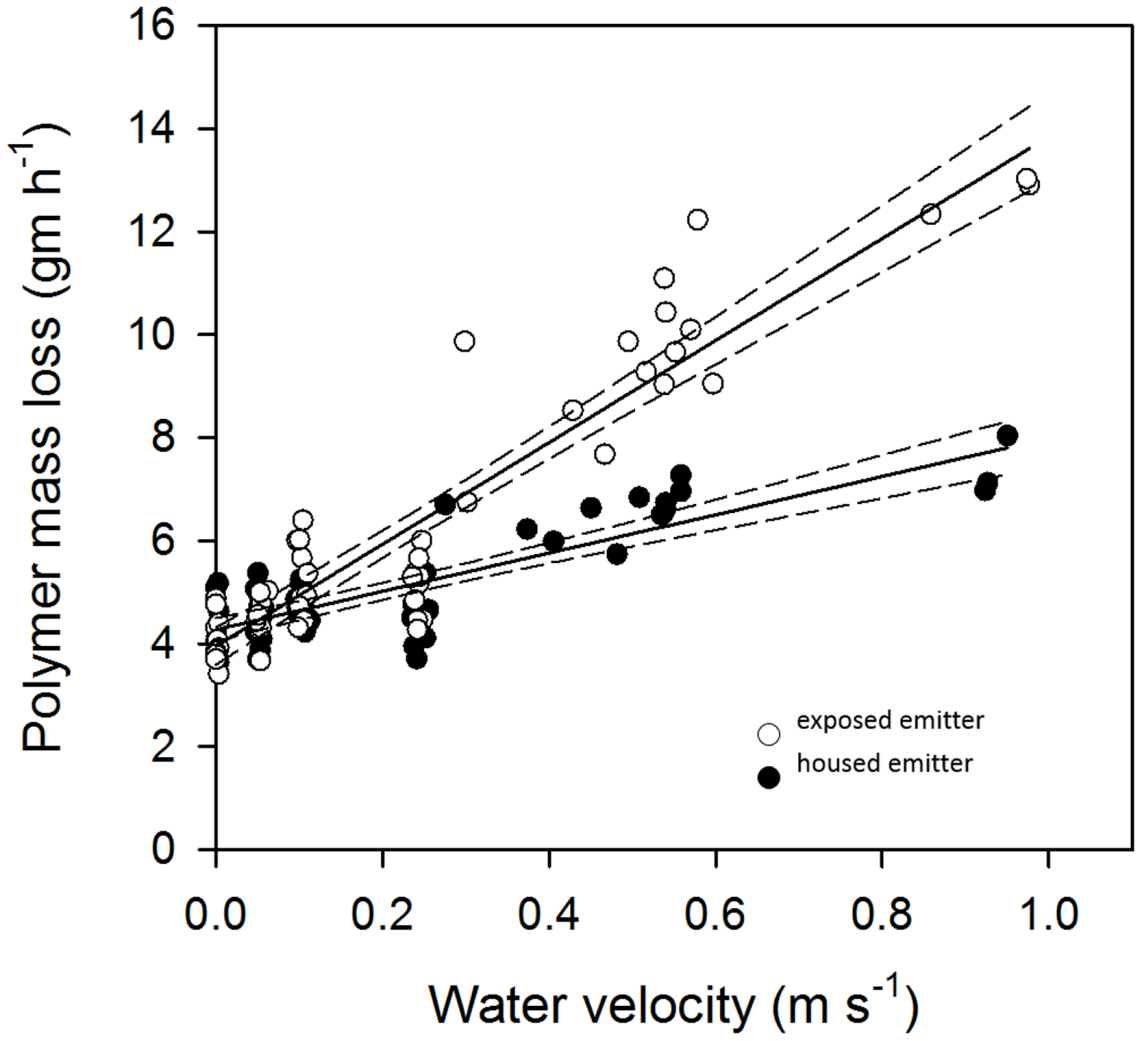
Housing the PEG6000 emitter better controlled the rate of dissolution at high water velocity. In a recirculating flume, increasing water velocity accelerated the rate of PEG6000 dissolution. However, housing the emitter (filled circles, r^2^ = 0.69) reduced the effect of velocity on the dissolution rate at velocities >0.25 m s^-1^ relative to emitters fully exposed to the flow (white circles, r^2^ = 0.87). Linear regression fits with 95% CI (dashed lines) are included to illustrate the patterns, see text for GLM results.

GLM (log likelihood = -170.566, AIC = 3.10) revealed significant effects of emitter type (z = -5.25, P< 0.001) and water velocity (z = 16.05, P< 0.001) on the rate of PEG6000 dissolution in a flowing tank. We illustrate the effects with linear regressions (Fig 7), where increasing water velocity increased the rate of dissolution for both treatments. Housing the emitter reduced the relative rate of loss by 62% (exposed emitter, r^2^ = 0.87, slope = 9.90 ± 1.04 (2SE); housed emitter, r^2^ = 0.69, slope = 3.72 ± 0.67 (2SE)). Visual examination of the raw data suggest the improvement in performance (stabilization of the dissolution rate) appeared at velocities >0.40 cm s^-1^.

## Discussion

Our findings validate polyethylene glycol as a useful material for the construction of inexpensive semiochemical emitters for use in natural waters. The behavioral reactivity of a partial sex pheromone was unaltered during PEG emitter construction, and controlled-release was achieved at a rate sufficient to be useful in conservation activities related to the management of invasive sea lamprey in the Great Lakes. Several destructive invasive fishes are known to rely on semiochemicals to complete critical aspects of their life cycle (e.g. bighead (*Hypophthalmichthys nobilis*) and silver (*H*. *molitrix*) carps [50], round goby (*Neogobius melanostomus*) [51, 52], brook trout (*Salvelinus fontinalis*) [53, 54]). As PEG is chemically inert and environmentally benign, similar emitters should prove useful in the deployment of semiochemicals to monitor and manage these species.

Exposure to PEG did not alter the behavior of migratory sea lamprey in the lab (Objective 1), and did not alter the response of ovulated females to a partial sex pheromone emitted from PEG in a natural stream (vs. pumped as a methanol-water mixture) (Objective 2). We conclude from these findings that the structure of the pheromone molecule was not significantly altered by the process of immersion and mixing in the melted polymer, and did not break down during short-term storage at room temperature, nor during the short-term field deployment. 3kPZS is a bile acid that exhibits significant stability when stored in dry form at -20 °C, and in natural stream waters has a nominal half-life of 26.1 h [55]. The breakdown of lamprey semiochemicals in streams, as with other organic molecules, is affected by microorganisms (bacteria, fungi, algae) and through adhesion to and/or absorption by particulate organic matter and biofilms [56]. Because PEG is not bioreactive and is not known to be consumed directly by microorganisms, biological breakdown of the molecule when entrained in the polymer is unlikely vs. other organic molecules that are similarly benign (e.g. starch). Thus, PEG may prove useful in the development of long acting emitters, reducing the effort necessary to maintain continued application. In addition, a cylindrical emitter, as used in this study, would allow for the creation of layers in the emitter where differing concentrations of the odorant were maintained (including blank layers). This would allow for targeted release during particular times of day (e.g. crepuscular periods) for emitters designed for multi-day deployments, and the ability to intentionally vary the concentration of the odor, avoiding issues with habituation in the responding organisms.

Baiting traps during control operations with the 3kPZS PEG6000 emitter (Objective 3) increased trapping success of sexually immature males and females, a finding consistent with previous studies utilizing pumped liquid mixtures of the pheromone. A large-scale field trial on 16 relatively small streams (discharge 0.5–8.0 m^3^ sec^-1^) found that when 3kPZS was pumped into a paired trap to reach a final in-stream concentration of 10^−12^ M, 54% of total catch was in the 3kPZS-baited trap [38], with ovulating females more likely to be captured in 3kPZS-baited traps. Here, baited traps captured 56% for all sexes and maturities combined and 75% of the ovulating females. A key difference between these studies, beyond using PEG emitters to apply 3kPZS, was that 3kPZS was applied at a fixed rate (2 mg h^-1^) rather than a rate that varied with discharge to achieve a fixed in-stream concentration of 10^−12^ M [38]. At an application rate of 2 mg h^-1^, our final instream concentrations ranged from 2.3–8.7 x 10^−14^ M. Application rates of roughly 20 to 300 mg h^-1^ would have been required to activate our study streams to 10^−12^ M. Therefore, these results support the hypothesis that in relatively large Great Lakes tributaries, controlled release of relatively small amounts of 3kPZS increase the probability that sea lamprey enter a trap as much as the previously applied larger amounts. Future research should seek to determine how dose influences the probability of encountering and/or entering the trap in different hydraulic conditions to facilitate matching the application protocol to the local circumstances [57–59]. In addition, government staff charged with operating the feeder device reported significantly reduced effort to manage the baiting of traps with polymer emitters vs. pumps, and the simple feeder device worked well in the varying weather conditions typical of spring and summer in the Great Lakes region.

Housing the emitter (Objective 4) significantly reduced the sensitivity of the dissolution rate to turbulence and high water velocity, though did not eliminate the effect altogether. The ability to withstand variation in discharge and velocity without altering emission rate is necessary to ensure controlled release is maintained for the intended duration. Though we partially achieved controlled release by housing the emitter, other solutions are apparent (e.g., timed-release of short duration emitters). However, the dispersion of semiochemicals in flowing water is subject to physical mixing via eddy diffusion and turbulence that can alter perception of the stimulus properties (ratio of odor components and/or the odor intensity) by decreasing the signal-to-noise ratio at distance from the source of emission [60]. Fishes and other aquatic organisms have sophisticated means to trace the varied concentrations encountered when swimming upstream to the source of an odor plume, and often exhibit attraction to off-mixture odor blends at distance from the point of emission [61, 62]. These capacities should ensure a PEG semiochemical emitter would remain effective as a lure despite uncontrolled variation in emission rate if the odor remains detectable to the sensory apparatus (i.e. above the minimum active concentration) over the intended area of effect.

## Conclusion

Semiochemicals, both attractive and repellent, hold considerable promise for the design of species-specific interventions to manage both destructive invasive taxa and species of conservation concern in aquatic ecosystems [6–10, 63]. Because the deployment of these odors is regulated in many jurisdictions, stable formulations of end-use products and well-defined application practices are required by law (e.g. the Federal Insecticide, Fungicide, and Rodenticide Act in the USA). PEG should readily conform to these requirements, as it is chemically inert, environmentally benign, and can be encapsulated in a degradable container (e.g. cardboard tubes) to avoid pollution concerns. Further, these cost-effective emitters should prove useful in scientific research into behavioral responses of aquatic organisms to varied numbers, spatial distributions, and intensities of odor sources, as the PEG matrix is unlikely to alter the chemical composition of the odorant(s) nor elicit a direct olfactory response from the animal.

## Supporting information

S1 Text Polymer evaluation and selection process. A description of the qualitative laboratory examinations of potential polymers that led to the selection of PEG6000.
(DOCX)

S1 Fig Typical laboratory release patterns of Rhodamine WT dye from three polymers. Observed dye release from polymers placed in the laboratory raceway for 12 h of flow. Data were taken at 5 second intervals. HEC = hydroxyethyl cellulose, PVA = polyvinyl alcohol, PEG = polyethylene glycol (MW = 6000).
(TIFF)
