# Supplementary material for: A simple, cost-effective emitter for controlled release of fish pheromones: Development, testing, and application to management of the invasive sea lamprey
Source: PLoS One. 2018 Jun 13;13(6):e0197569. doi: 10.1371/journal.pone.0197569 (PMC5999092; doi:10.1371/journal.pone.0197569)
Supplement: S1 Text — A description of the qualitative laboratory examinations of potential polymers that led to the selection of PEG6000. (DOCX) [file pone.0197569.s001.docx]

**Supplemental Information – Polymer Evaluation and Selection Process**

Five polymers were selected for initial evaluation after applying two criteria: (1) each candidate must be cost-effective to produce and use, equating to high commercial availability and relatively low cost; and, (2) each candidate must be environmentally benign (i.e. was included on the U.S. Environmental Protection Agency’s schedule of exempt polymers per the U.S. code of federal regulations, 40.CFR.180). The polymers chosen for initial evaluation were the natural or naturally derived polymers starch, pullulan, and hydroxyethyl cellulose (HEC), and the synthetic polymers PVA and PEG (five molecular weights, 2000, 4000, 6000, 8000 and 10,000 g mol^-1^). We constructed test emitters by melting each polymer and pouring it into small PVC tubes (5 cm L, 1.3 cm ID) to cool and solidify. We examined the following properties of each polymer in the test emitter configuration.

*Melting Point:* To prepare the emitters, 3kPZS would be blended into molten polymer. 3kPZS melts at 172-180 °C (Bridge Organics, Inc.); thus, we desired a polymer that would melt below 160 °C to ensure the process of emitter construction would not degrade the pheromone. PEG exhibited the lowest melting point (50-100 °C depending on molecular weight). The pure melting points of the other polymers were potentially too high: PVA melts between 180-250 °C (depending on the degree of hydrolysis), and the carbohydrate polymers (starch, pullulan and HEC) melt above 250 °C. However, it is known that starch and PVA can be plasticized by glycerol, lowering their melting points. By heating different blends of these polymers with glycerol, we reduced their melting points to <120 °C.

*Dissolution behavior in water:* The polymer must readily dissolve in water at a steady rate, ensuring controlled release of the pheromone over the course of 9-12 hours (i.e. a single night, as the sea lamprey is predominantly nocturnal). Polymer dissolution behavior was examined using three tests. First, nominal mass loss (dissolution rate) was estimated by placing each emitter into a 2 L flask with 1 L of continuously stirred 10 °C water (a temperature typical of Great Lakes streams during the sea lamprey migration). Every 30 min the water was replaced. From each 30 min aliquot, a 10 mL sample was collected and evaporated, and the polymer residue was weighed on an analytical balance. Second, new emitters were formed from each polymer mixed with a tracer dye (Rhodamine WT, as a concentrated solution of 1 mg μl^-1^ in methanol) and examined in two laboratory tests. *Test #1:* The rate of dye release was estimated in the laboratory in 2 L flasks following the methods for the mass loss estimate. Here, the dye concentration for each 30 min sample was determined via UV/V spectroscopy using an Olis HP 8452 Diode Array Spectrophotometer; Rhodamine WT exhibits a UV maximum at 530 nm. The rate of dye release was estimated by comparison of the absorbance at 530 nm to a calibration curve to determine the Molar concentration. The results of these preliminary laboratory tests were used to prepare emitters with sufficient dye loading for the second test. *Test #2:* We constructed a small (0.25 m wide, 0.125 m deep, 3.05 m long) semi-circular PVC flow channel at the U.S. Geological Survey’s Hammond Bay Biological Station (HBBS) near Millersburg, Michigan, USA in July of 2010. Water was pumped into the channel from Lake Huron at a rate of 22 L min^-1^ generating an average observed velocity of 2.27 cm s^-1^. Dye concentration was monitored at 5-second intervals with a continuously recording sonde (Turner Designs, Inc. DataBank with a Cyclops Rhodamine submersible sensor). A single emitter was suspended perpendicular to the flow at mid-depth and dye release was monitored for 12 h. Five trials were completed for each emitter type.

In the initial laboratory tests (Test #1) both starch (<2 h) and pullulan (6-8 h) dissolved too quickly and exhibited inconsistent dye release behavior, and were eliminated from further consideration. Both HEC and PVA dissolved slowly (≈12 h); however, whereas dye release from HEC tended to diminish after 3 h in the flow channel, the PVA dissolved released dye at a steady rate (Fig. 1). The PEG emitters dissolved over a range of times reflecting their molecular weights: the 2000 molecular weight took ≈2 h, the 4000 ≈4 h, 6000 and 8000 molecular weights took 5-6 h, while the 10000 molecular weight took >8 h. During the flow tank trials (Test #2), the emitters varied in size based on the observed dissolution rate in the laboratory (i.e. the length and diameter of the tubes were configured to last ~ 12 h). The PEG emitters released dye at a steady rate during dissolution. (Fig. 1). HEC, PVA and PEG met the basic performance standards for use as a fish pheromone emitter (>8 h dissolution time and controlled release of dye). However, both PVA and HEC exhibited relatively high melting points and require plasticization with glycerol prior to emitter construction. At the time of the study, HEC cost ~$200 (US) per kg, PVA ~$125 per kg, and PEG $50-100 per kg depending on molecular weight. Based on cost, ease of construction, low melting point, and availability, we choose PEG at a molecular weight of 6000 g mol^-1^ for behavioral testing with 3kPZS.
