# Supplementary figures and images for: A simple, cost-effective emitter for controlled release of fish pheromones: Development, testing, and application to management of the invasive sea lamprey
Source: PLoS One. 2018 Jun 13;13(6):e0197569. doi: 10.1371/journal.pone.0197569 (PMC5999092; doi:10.1371/journal.pone.0197569)

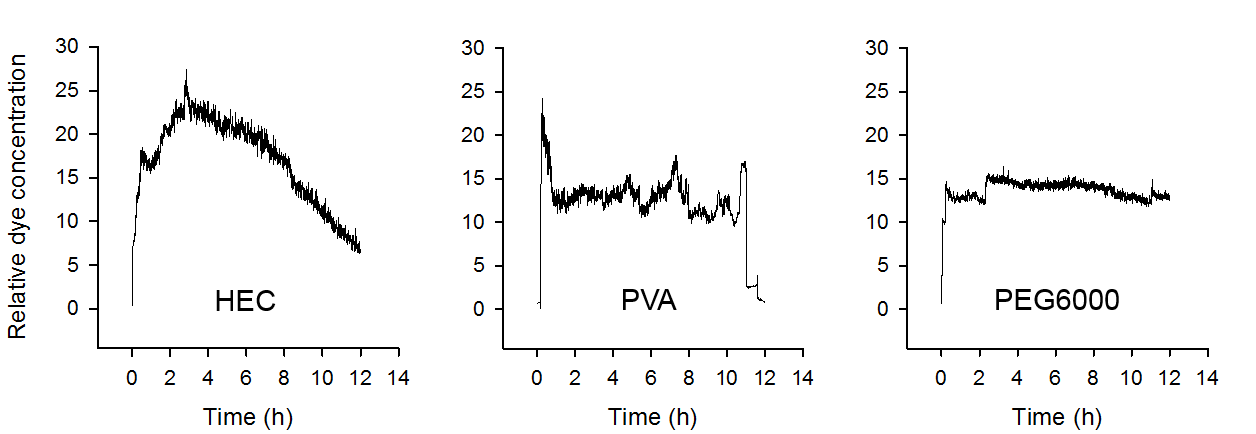

Supplement: S1 Fig — Observed dye release from polymers placed in the laboratory raceway for 12 h of flow. Data were taken at 5 second intervals. HEC = hydroxyethyl cellulose, PVA = polyvinyl alcohol, PEG = polyethylene glycol (MW = 6000). (TIFF) [file pone.0197569.s002.tiff]
